# Logistic and organizational aspects of a dedicated intensive care unit for COVID-19 patients

**DOI:** 10.1186/s13054-020-02955-x

**Published:** 2020-05-18

**Authors:** Maria Vargas, Giuseppe De Marco, Stefania De Simone, Giuseppe Servillo

**Affiliations:** 1 Department of Neurosciences, Reproductive and Odontostomatological Sciences, University of Naples “Federico II”, Via Pansini, 80100 Naples, Italy; 2 Institute for Research on Innovation and Services for Development, National Research Council of Italy, Via San Felice, Naples, Italy

Dear Editor,

On 31 March, the World Health Organization (WHO) reported 750,890 confirmed globally confirmed cases of COVID-19 [1]. COVID-19 cases are dramatically increasing in several countries with heterogenous and unpredictable distributions [2]. Patients with COVID-19 have resulted in high rates of hospitalization and ICU admissions [3]. On 6 April, the worldwide ICU admission rate of COVID-19 patients was 3% ranging from 1% of total cases in Africa to 4% of total cases in Europe [4]. According to this heterogenous and unpredictable geographic distribution of COVID-19, several countries are increasing their ICU capacity response by converting general ICU in dedicated COVID-19 facilities [5]. Dedicated ICUs for COVID-19 patients were suddenly created on the whole Italian territory [5]. Based on the high contagiousness of the 2019 novel CoV virus [6], the logistics and the staff organizations are the fundamental principles to avoid the in-hospital spread of the virus while creating dedicated COVID-19 facilities. From 10 March, our ICU is completely dedicated to COVID-19 patients, and actually, it is one of the largest cohorted ICU in the south of Italy admitting 12 positive critically ill patients (Fig. 1). The ICU is divided into green, yellow, and red areas (Fig. 1). Each ICU bed is equipped with a full monitoring of vital parameters and a mechanical ventilator. Each monitor is duplicated in the centralized control unit equipped with microphones and glasses to allow the communications between the staff. Inside the ICU, we have a laboratory section including two dedicated ultrasound machines, disposable fiberoptic bronchoscopes, video laryngoscopes, point-of-care arterial blood gas and coagulation analyses, transport ventilator, and emergency cart with a defibrillator.

**Fig. 1:**
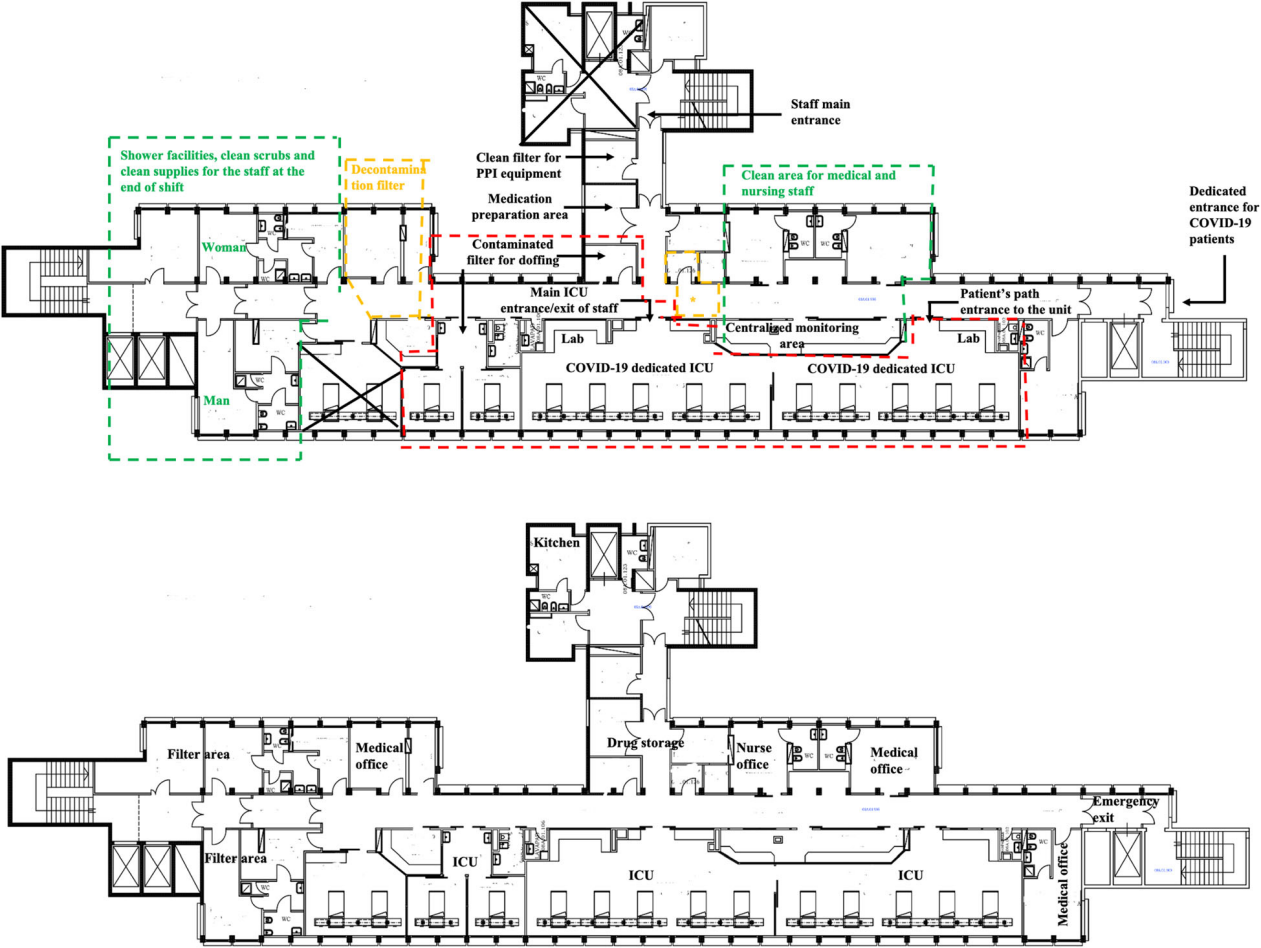
Upper box—organization of ICU before COVID-19 outbreak. Before the outbreak of COVID-19, our ICU was equipped with two main boxes including 5 beds each and one box with two beds for the infected patients. We also had several offices for medical and nursing staff. Lower box—organization of ICU dedicated to COVID-19 patients. The red area is a zone where the full PPE is mandatory. The yellow areas are the zone of decontamination while the green area is a clean one. The entrance of the first ICU box was the only entry point for COVID-19 patients. The green area inside the ICU is a clean zone where the medical and nursing staff may stay during the 12-h shift. The green area outside the ICU, equipped with shower facilities, clean scrubs, and clean supplies, is dedicated to the staff wash at the end of shift. The yellow areas are the filter of decontamination where the staff must change their scrubs, wash their body with disinfectants, and clean their shoes in the bowls with sodium hypochlorite 0.1 to 0.5 before accessing the green areas. Inside the red area, we set up two contamination filters, equipped with waste management material, mirror, and supply to wash the body and the hand, for doffing after the exiting from the ICU boxes

During the 12-h shift, the nursing and medical working is organized as follow:

1. The most experienced ICU physician is the work shift coordinator and stays in the green area to control the compliance of the staff with the procedures and to check the patients from the centralized monitoring area.
2. Medical staff review the medical records of each patient, and then a briefing with the whole staff is made to plan the actions of the shift.
3. All the therapies are prepared in a dedicated area outside the ICU boxes to minimize the time spent inside.
4. The nursing and medical staff performed the first entry in the ICU boxes and stay inside for 4 h. After that, only two nurses and one medical doctor continue to stay inside for an additional 2 h while the other personnel rest themselves and fulfill the medical records.
5. After that, the nurses and the medical doctor are replaced by other 3 colleagues for another 2 h.
6. The transition from the red to the green area must be preceded by the staff decontamination in the yellow area.
7. The disinfection of the different areas is performed three times during the shift.

According to our experience, a simple logistic project and clear organizational plan may be the keys to the success of surging the ICU capacity with dedicated facilities during the COVID-19 outbreak.

## Data Availability

NA
